# Suppression of Tumor Aggression Through Metabolic Reprogramming via Oxamate Targeting LDHA

**DOI:** 10.3390/ijms27073245

**Published:** 2026-04-02

**Authors:** Yurii V. Stepanov, Galyna I. Solyanik, Yulia Yakshibaeva, Denis Kolesnik, Liudmyla I. Stepanova, Iuliia Golovynska

**Affiliations:** 1 Department of Pathophysiology of Metastasis, R.E. Kavetsky Institute of Experimental Pathology, Oncology and Radiobiology, National Academy of Sciences of Ukraine, 03022 Kyiv, Ukraine; lestehprom@gmail.com (Y.V.S.); gsolyanik@gmail.com (G.I.S.); yakshibaeva@gmail.com (Y.Y.); denkolesnik83@gmail.com (D.K.); 2 Institute of Biology and Medicine, Taras Shevchenko National University of Kyiv, 01601 Kyiv, Ukraine; ulunga@ukr.net; 3 Key Laboratory of Optoelectronic Devices and Systems, College of Physics and Optoelectronic Engineering, Shenzhen University, Shenzhen 518060, China

**Keywords:** lactate dehydrogenase, circulating tumor cells, cancer metabolism, blockade of glycolysis, sodium oxamate, anoikis resistance, migration, invasion, metastasis

## Abstract

Lactate dehydrogenase (LDH) is a key glycolytic enzyme that catalyzes the interconversion of pyruvate and lactate, with LDHA gaining particular attention for its overexpression in various malignancies and pivotal role in the Warburg effect-driven metabolic reprogramming. Elevated LDHA activity supports rapid ATP production under hypoxic conditions, maintains NAD^+^ regeneration, and promotes lactate accumulation, creating an acidic tumor microenvironment (TME) that favors invasion and immune evasion. Accumulating evidence demonstrates that LDHA is essential for primary tumor growth and critically involved in circulating tumor cell (CTC) survival, anoikis resistance, and metastatic spread. These functions are mediated by its regulation of adhesion molecules, cytoskeletal remodeling, and energy adaptation that enable CTCs to withstand mechanical shear stress and immune surveillance in the bloodstream. Pharmacological inhibition of LDHA, particularly via sodium oxamate (oxamate), has shown substantial potential in reducing metastasis and enhancing chemotherapy sensitivity in preclinical models. Oxamate has emerged as a promising candidate for metabolic cancer therapy due to its unique double effects on tumor metabolism and anti-tumor immunity, which are an advantage rarely highlighted in broader LDHA-focused reviews. This review synthesizes the molecular mechanisms through which LDHA drives tumor progression, dissects its context-specific functions in CTC biology, and evaluates the translational potential of LDHA-targeted strategies, with a focused emphasis on oxamate, as a transformative anti-metastatic therapeutic paradigm. By filling a critical gap in synthesizing oxamate’s distinct metabolic–immune regulatory actions, this work addresses an unmet need in the management of advanced, treatment-refractory cancers.

## 1. Introduction

Metabolic reprogramming is a defining feature of malignant transformation, enabling cancer cells to sustain uncontrolled growth and survive in hostile microenvironments [1]. One of the most prominent metabolic adaptations is the preferential conversion of glucose to lactate, even in the presence of oxygen, known as the Warburg effect [2,3]. This shift provides not only a quick way to generate adenosine triphosphate (ATP) but also a constant supply of the glycolytic intermediates needed for biosynthesis and redox balance. At the core of this process is lactate dehydrogenase (LDH), a crucial enzyme that links cytosolic glycolysis with mitochondrial metabolism by catalyzing the reversible conversion of pyruvate to lactate and regenerating NAD^+^ [4,5,6]. This metabolic switch allows cancer cells to acquire the necessary building blocks and energy to support their survival, resist hypoxia and treatment, grow rapidly, and promote cell migration and invasion. It is also important to note that high levels of LDH are often associated with poor prognosis in various human cancers, highlighting its potential role in disease progression [7,8,9,10].

LDH exists as several isoenzymes composed of different subunits (LDHA and LDHB), with their levels varying across tissues and tumor types [11]. Among these, LDHA has gained particular attention because of its consistent upregulation in aggressive and poorly differentiated cancers. Increased LDHA activity leads to lactate buildup, reduces intracellular pyruvate availability for mitochondrial oxidation, and thus supports the glycolytic phenotype that drives tumor growth and adaptation to hypoxia. Additionally, lactate, once thought to be just a metabolic byproduct, has become a key signaling molecule within the tumor microenvironment (TME). It influences gene expression, angiogenesis, and immune evasion by acidifying the environment and through paracrine signaling, creating a permissive niche for invasion and metastasis [12].

Beyond its metabolic function, LDH plays a role in several cancer hallmarks. Recent data show that LDHA supports epithelial–mesenchymal transition (EMT), enhances cytoskeletal flexibility, and facilitates tumor cell detachment and movement. These processes are vital for the creation and survival of circulating tumor cells (CTCs), which serve as seeds for metastatic spread [4]. LDH-mediated control of redox equilibrium and energy homeostasis is a critical adaptive mechanism that endows CTCs with resistance to oxidative and mechanical stress during hematogenous dissemination [13]. Simultaneously, lactate-rich environments induced by LDH overactivity suppress anti-tumor immune responses, particularly by inhibiting cytotoxic T lymphocytes (CTLs) and natural killer (NK) cell functions, thereby further enhancing metastatic success [14,15,16].

The therapeutic relevance of LDH has become increasingly evident. Pharmacological or genetic inhibition of LDHA disrupts glycolytic flux, lowers lactate production, and restores mitochondrial oxidative phosphorylation (OXPHOS). These changes lead to suppressed growth, increased reactive oxygen species (ROS) generation, and greater sensitivity to chemotherapeutic drugs in various preclinical cancer models. Small-molecule inhibitors like sodium oxamate (oxamate), galloflavin, FX11, and GNE-140 have been extensively researched for their ability to selectively block LDH-driven metabolic adaptation [4,17,18,19,20]. Although translating these inhibitors into clinical use remains difficult due to issues with specificity and pharmacokinetics, their success in experimental systems highlights LDH as a promising target for metabolic intervention. Given the multifaceted contribution of LDH to tumor energy production, redox balance, and metastatic ability, understanding how it mechanistically fits into cancer metabolism holds significant translational potential. An increasing number of studies emphasize not only its enzymatic function but also its regulatory interactions with oncogenic signaling pathways such as MYC, hypoxia-inducible factor 1 alpha (HIF-1α), and PI3K/AKT/mTOR. These pathways work together to maintain LDHA levels and support the glycolytic phenotype during stress conditions [21,22,23,24,25].

We prioritized oxamate as a prototypical LDHA inhibitor due to the substantial body of experimental evidence supporting its biological effects following LDHA blockade [4,18,19,26,27,28,29,30,31,32,33,34,35,36,37,38,39,40,41,42,43,44,45,46]. The synthesis of these studies in the present review enables a comprehensive evaluation of the metabolic consequences associated with LDHA inhibition and their anticancer implications. Although alternative inhibitors (e.g., GSK2837808A) exhibit greater potency in vitro, their preclinical characterization remains limited. Notably, despite its comparatively lower in vitro activity, oxamate demonstrates superior antitumor efficacy in vivo, likely attributable to pharmacokinetic properties rather than fundamental differences in LDHA-targeting mechanisms [39,47].

Among small-molecule inhibitors targeting LDHA, oxamate distinguishes itself through its underappreciated and multifaceted potential, which sets it apart from counterparts like galloflavin or FX11. Oxamate, by blocking LDHA-mediated glycolysis, alters the TME, neutralizing lactate-induced immunosuppression. This metabolic–immune modulation addresses a critical unmet clinical need in advanced, treatment-refractory cancers, where single-mechanism therapies often fail to overcome the interconnected barriers of metabolic plasticity and immune evasion. Notably, this review fills a key gap in existing LDHA-focused syntheses by centering on oxamate’s distinct role in suppressing metastatic progression through metabolic–immune crosstalk, which is a topic remained underexplored in prior literature.

Building on this premise, this review aims to systematically consolidate the current understanding of the molecular mechanisms by which LDHA orchestrates cancer cell metabolic reprogramming, with a deliberate focus on its non-negligible role in sustaining CTC survival, resisting anoikis, and facilitating metastatic dissemination. Beyond dissecting these mechanistic underpinnings, the review explores emerging therapeutic strategies targeting LDHA activity, with special emphasis on oxamate, and critically assesses the translational potential of combining LDHA inhibition with conventional chemotherapeutics, immunotherapies, and immunometabolic agents. By integrating preclinical evidence across diverse cancer models, this work seeks to validate a rational, combinatorial approach to attenuate tumor spread and improve outcomes for patients with aggressive, metastatic disease.

## 2. Metabolic Regulation and the Function of LDHA in Cancer Cells

### 2.1. LDHA as a Key Factor in Cancer Metabolism Reprogramming

**Figure 1:**
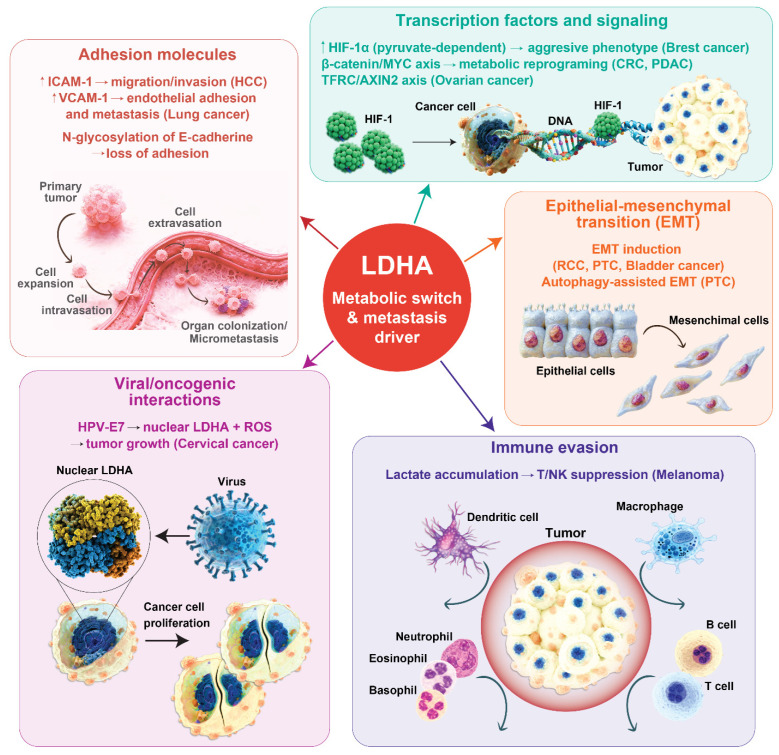
LDHA functions as a convergent regulator of metastatic progression across diverse cancer types. Schematic illustrating the integrated molecular mechanisms by which LDHA drives metastatic progression in distinct tumor contexts. LDHA-mediated enhancement of glycolytic flux and lactate production supports multiple pro-metastatic processes: induction of EMT, modulation of adhesion molecules (including ICAM-1, VCAM-1, and E-cadherin glycosylation), stabilization of HIF1α, metabolic rewiring, and immune evasion via TME acidification by accumulated lactate. Tumor-type-specific regulatory pathways are also depicted, underscoring the role of LDHA as a conserved mediator of cancer cell invasion, migration, and metastatic dissemination.

LDHA overexpression is one of the most consistent metabolic changes seen in malignant tumors and is increasingly recognized as a functional driver of cancer development rather than just a passive biomarker [48,49,50]. It is observed across various tumor types, including papillary non-small-cell lung cancer (NSCLC) [51], papillary thyroid carcinoma (PTC) [52], nasopharyngeal carcinoma (NPC) [53,54], esophageal [18], gastric [31,55], pancreatic [56,57,58,59], hepatocellular carcinoma (HCC) [33,60], colorectal cancer (CRC) [61], renal cell carcinoma (RCC) [36], muscle invasive bladder cancer (MIBC) [62,63], endometrial cancer [64], melanoma [65], cervical tumor [8], ovarian cancer [66], breast cancer [67], prostate cancer [68], glioblastoma (GBM) [69], and other [45,70] (Table 1). Elevated LDHA levels are linked to aggressive tumor behavior, resistance to therapy, and poor patient outcomes. While elevated LDHA expression correlates with aggressive tumor phenotypes and is a surrogate marker for heightened glycolytic flux in cancer cells, LDHA is a catalytic driver of the aerobic glycolysis that underpins malignant progression. Indeed, aerobic glycolysis (the Warburg effect) in cancer cells is functionally dependent on LDHA activity, with its inhibition ablating this hallmark metabolic reprogramming and attenuating tumor aggressiveness [71,72]. The widespread presence of LDHA in different types of cancers highlights its critical role in metabolic reprogramming, as it is a hallmark of cancer that helps support rapid biomass production and adaptation to stress in the microenvironment [70,73]. However, the glycolytic effects of LDHA overexpression stand in direct contrast to those of exogenous lactate supplementation in cancer cells. Whereas LDHA overexpression drives enhanced glycolytic flux by accelerating pyruvate-to-lactate conversion and sustaining NAD^+^ regeneration to fuel unremitting glycolysis [48], exogenous lactate supplementation conversely suppresses glycolytic activity via distinct feedback mechanisms. Exogenous lactate elevates intracellular lactate/pyruvate ratios, reducing NAD^+^ bioavailability and directly inhibiting key glycolytic enzymes; additionally, lactate uptake via monocarboxylate transporters (MCTs) shifts cancer cell metabolism toward oxidative phosphorylation and modulates glycolytic gene expression through the activation of signaling cascades including HIF1α [74,75]. This divergence in metabolic regulation highlights the nuanced, context-dependent role of lactate metabolism in cancer, wherein LDHA-mediated lactate production sustains the Warburg effect, while extracellular lactate uptake induces a compensatory metabolic shift that tempers glycolytic activity [76]. Notably, high LDHA expression additionally endows tumor cells with the capacity to scavenge and utilize extracellular lactate as a metabolic fuel. The enzyme catalyzes the reverse conversion of lactate back to pyruvate, which can be further converted to oxaloacetate via pyruvate carboxylase to feed gluconeogenic pathways, thereby furnishing de novo glucose to sustain glycolytic flux even in nutrient-limited TMEs [77,78]. Therefore, LDHA functions as a key component of metabolic adaptation, promoting the aggressive nature of tumor cells (Figure 1). To sustain this glycolytic and pro-metastatic phenotype, these cells upregulate LDHA through various oncogenic pathways, including HIF1α stabilization under hypoxia, MYC-driven transcriptional induction, PI3K/AKT/mTOR activation, KRAS signaling, and epigenetic mechanisms like promoter demethylation [22,23,24,25]. This convergence of regulation ensures that rapidly dividing cancer cells maintain a steady supply of NAD^+^ and ATP, even when mitochondrial respiration is impaired or intentionally reduced [79]. Elevated LDHA promotes tumor progression via several interconnected mechanisms. First, increased lactate production acidifies the extracellular environment, supporting local invasion by enhancing matrix breakdown, modulating integrin signaling, and facilitating EMT [80]. Acidification also suppresses the activity of CTLs and NK cells, creating an immunosuppressive microenvironment that permits immune escape [81]. Second, LDHA helps maintain redox balance by regulating the NAD^+^/NADH ratio, allowing cancer cells to buffer oxidative stress and survive conditions of detachment, hypoxia, or nutrient deprivation. This is especially important for CTCs, whose survival during dissemination relies heavily on LDHA-driven metabolic flexibility [82].

Moreover, LDHA overexpression is linked to a high metastatic potential (Table 1) (Figure 1). In many models, such as triple-negative breast cancer, non-small cell lung cancer, HCC, and melanoma, genetic knockdown or pharmacological inhibition of LDHA decreases metastatic spread by impairing cell motility, cytoskeletal dynamics, and resistance to anoikis [60,83]. Lactate itself functions as an autocrine and paracrine signaling molecule, activating pathways like NF-κB, GPR81, and TGF-β, which further promote EMT programs and stromal reprogramming [84]. Tumor-associated fibroblasts and endothelial cells also respond to lactate, enhancing angiogenesis and remodeling the TME [81,85]. High LDHA expression has important therapeutic implications. Many preclinical studies demonstrate that LDHA inhibition, through agents such as oxamate, FX11, galloflavin, GNE-140, or genetic silencing, causes an energetic crisis, increases ROS accumulation, sensitizes tumors to radiotherapy and chemotherapy, and reduces invasive and metastatic traits [26,72,86,87,88,89].

In summary, elevated LDHA is not just a metabolic signature but a key driver of tumor progression (Figure 1), coordinating bioenergetics, redox balance, metastatic ability, and immune evasion. Its widespread upregulation across various cancer types underscores LDHA as a promising target for metabolic treatments and combination therapies designed to inhibit tumor growth and spread.

### 2.2. Regulation of LDHA by Transcription Factors

Available data indicate that LDHA may possess non-canonical functions beyond its established metabolic role. Early studies demonstrated that LDHA can bind AU-rich elements within GM-CSF mRNA and interact with AUF1, an RNA-binding protein involved in mRNA stability [2]. Additionally, LDHA has been reported to associate with translationally active polysomes and to enhance DNA synthesis mediated by the DNA polymerase-α complex [2,3]. Notably, tyrosine-phosphorylated LDHA has been detected in the nucleus, implying a potential regulatory role linked to post-translational modification [4]. Collectively, these findings raise the possibility that LDHA participates in gene regulatory processes; however, the precise mechanisms remain poorly defined, and specific transcriptional targets have yet to be clearly established. Given that much of this evidence originates from earlier studies, further investigation using modern molecular and genomic approaches is warranted to clarify these non-metabolic functions.

More recent investigations have revisited these observations using modern molecular approaches. Current evidence indicates that, although LDHA does not function as a classical transcription factor, it may indirectly influence transcriptional programs through metabolite-dependent chromatin modifications and signaling crosstalk. For instance, lactate produced by LDHA has been shown to drive histone lactylation (HKla), thereby modulating gene expression in a context-dependent manner [1,90]. In addition, recent reviews and experimental studies highlight that lactate acts as both a metabolic intermediate and a signaling molecule shaping transcriptional responses within the TME [5,6]. Importantly, genome-wide and proteomic analyses performed in recent years have not identified sequence-specific DNA binding by LDHA, arguing against a direct transcription factor-like role. Instead, LDHA-dependent metabolic flux has been linked to regulation of chromatin accessibility and histone modifications, including acetylation and lactylation, thereby indirectly influencing gene expression programs [7]. Furthermore, recent work suggests that nuclear localization of LDHA, particularly under oncogenic or stress conditions, may contribute to gene regulation through protein–protein interactions rather than direct DNA binding [8,9]. These findings support a model in which LDHA acts as a metabolic regulator of transcriptional output, integrating cellular metabolic state with epigenetic and signaling networks.

Despite these advances, definitive evidence for a direct transcriptional role of LDHA remains lacking. Thus, while historical studies point to potential RNA- and chromatin-associated functions, contemporary data largely support an indirect, metabolism-driven mechanism of gene regulation.

The promoter region of the LDHA gene contains multiple binding sites for various transcription factors, indicating the potential for complex regulation of LDHA expression. LDHA plays a key role in the transformation process driven by the transcription factor c-Myc. For c-Myc to effectively transform normal cells into tumor cells, it must activate LDHA expression [91]. The Myc family of proto-oncogenes is crucial in developing many cancer types, acting as an important oncogene after mutation. The proto-oncogene c-Myc can form a heterodimer with the Myc-associated factor X (MAX). This heterodimer binds to the E-box in the LDHA promoter, thereby activating gene transcription [91]. Notably, there is a positive correlation between the expression levels of c-Myc and LDHA in pancreatic cancer cells. Suppressing c-Myc expression leads to decreased LDHA levels, which reduces lactate production and glucose consumption [22]. Conversely, inhibiting LDHA causes an increase in c-Myc expression, demonstrating a negative feedback mechanism by which LDHA regulates c-Myc levels. The synergistic interaction between LDHA and Rcl, one of the target genes of c-Myc, supports growth induction independent of attachment [92], emphasizing the role of LDHA in cancer development and progression.

The dependence of LDHA transcription on the transcription factor heat-shock factor 1 (HSF1) has been demonstrated. HSF1 plays a central role in maintaining proteostasis in response to stress [93,94]. It is important to note that HSF1 also functions as a transcription factor regulating LDHA expression. Studies have shown that the ErbB2 oncogene can elevate HSF1 levels [95]. Simultaneously, an increased level of HSF1 is found at the promoter region of the LDHA gene, leading to heightened LDHA expression. This underscores the vital connection between HSF1 and LDHA, highlighting the significant role of LDHA in the cell’s metabolic adaptation [95]. This mechanism may have important implications for understanding how cells adapt to various stress conditions and for developing new therapeutic strategies aimed at modulating HSF1 activity and, consequently, LDHA expression.

It has been shown that LDHA expression is regulated by oxygen levels, controlled by HIF1 [96]. Examination of the LDHA gene’s promoter region revealed two functional HIF1 binding sites, known as hypoxia response elements (HREs) [96]. A positive correlation between LDHA levels and HIF1α is observed in NSCLC cells [97]. This suggests that LDHA might be key in the tumor’s adaptive response to hypoxia. Additional studies confirmed that HIF1α directly controls LDHA expression by binding to the HRE in the gene’s promoter region [96]. Therefore, under hypoxic conditions, HIF1 and HIF1α activate LDHA transcription, increasing enzyme levels in the cell. Furthermore, LDHA regulation in cancer cells is even more complex. It has been shown that the c-Myc oncogene and HIF1α can work together to activate LDHA transcription in various tumor types [98]. This synergy underscores the significance of LDHA in preserving the metabolic properties of cancer cells, thereby supporting their survival and growth.

Attention has been paid to the role of Krüppel-like factor 4 (KLF4) in regulating metabolism, particularly its effect on LDHA expression. The relationship between KLF4 and LDHA represents a key intersection between metabolic and transformational programs in cancer cells. Acting as a tumor suppressor, KLF4 directly represses LDHA gene transcription by binding to its promoter, leading to reduced glycolytic activity and decreased lactate production [57]. Simultaneously, KLF4 inhibits caveolin-1 expression, limiting PI3K/AKT/mTOR-dependent LDHA stabilization and blocking EMT processes. In contrast, the loss of KLF4 enhances LDHA expression, promotes a metabolic shift toward aerobic glycolysis, and stimulates an invasive phenotype [99]. Thus, KLF4 functions as a coordinator of the energetic and transformational status of tumor cells, restraining cancer progression and metastasis through transcriptional and signaling suppression of LDHA-dependent Warburg metabolism.

Current evidence does not directly demonstrate coordinated cross-regulatory crosstalk between MYC/MAX, SP1, and KLF family members at the LDHA promoter. However, indirect evidence supports the biological plausibility of such interactions. c-Myc/MAX is a well-characterized transcriptional activator of LDHA [100], SP1 regulates glycolytic genes via GC-rich promoter motifs [101], and KLF4, which binds identical GC-rich elements, represses LDHA transcription [57]. Notably, MYC, SP1, and KLFs exhibit functional interplay at other gene loci, competing or cooperating for promoter binding to modulate transcription. Thus, while direct LDHA-specific crosstalk remains unproven, overlapping DNA-binding specificities and conserved regulatory interactions support the potential for competitive or cooperative LDHA regulation, a possibility that warrants acknowledgment.

Evidence suggests that the transcription factor cyclic adenosine monophosphate (cAMP) response element-binding protein (CREB) may regulate LDHA. Two strong observations support this idea. CREB targets the cAMP-dependent protein kinase A (cAMP-PKA) signaling pathway, which is activated through phosphorylation [102]. This pathway often responds to various cellular stresses and environmental changes, making CREB sensitive to the cell’s metabolic needs. Importantly, the conserved sequence CRE is found in the promoter region of the LDHA gene. This sequence is known to be a binding site for CREB [103]. Its presence indicates that CREB can directly bind to DNA near the LDHA gene and control its transcription. Together, these findings suggest that activating CREB via the cAMP-PKA pathway may allow CREB to bind to the CRE in the LDHA promoter and regulate LDHA gene expression. This mechanism could be crucial for how cells adjust their energy metabolism in response to environmental changes and metabolic demands.

The crucial role of the forkhead box M1 (FOXM1)-LDHA axis in the metabolic reprogramming of cancer cells is highlighted [104]. In pancreatic and gastric tumors, a positive correlation between FOXM1 and LDHA expression levels was observed. FOXM1 directly binds to the LDHA promoter, stimulating its transcription and resulting in increased LDHA activity [24,48]. Therefore, by regulating LDHA expression, FOXM1 plays a significant role in the metabolic plasticity of cancer cells, supplying them with the energy and building blocks needed for rapid growth and metastasis.

LDHA is recognized as a key component in the metabolic adaptation of cells to stressful conditions or those demanding increased glycolysis. 12-O-tetradecanoylphorbol-13-acetate (TPA), a well-known activator of protein kinase C (PKC), initiates a series of signaling events leading to the phosphorylation of the transcription factor activator protein-1 (AP1) [105]. Activated AP1 then binds to the TPA-responsive element (TRE) in the promoter regions of target genes, triggering their transcription. In terms of metabolic regulation, the LDHA gene, a crucial enzyme in glycolysis, is of particular interest. Notably, the promoter region of the LDHA gene contains TRE, indicating that LDHA can be a direct target for AP1 regulation activated by TPA. In essence, TPA, via PKC activation and subsequent AP1 phosphorylation, can directly influence the expression of LDHA [105,106]. Highlighting LDHA emphasizes the role of AP1 activation under TPA influence as a significant regulator of cellular metabolic adaptation.

## 3. Post-Translational Regulation of LDHA

### 3.1. Phosphorylation: Enhancement of LDHA Activity and Cancer Progression

Phosphorylation of specific amino acid residues in LDHA can substantially increase its enzymatic activity, which is often associated with cancer progression. For instance, in breast cancer cells [107] and CRC [108], phosphorylation of tyrosine at position 10 (Y10) activates LDHA, promoting cancer cell motility and resistance to anoikis. These studies revealed a positive correlation between the expression of the human coilin-interacting nuclear ATPase protein (hCINAP) and LDHA phosphorylation at Y10. The mechanism of hCINAP involves binding to LDHA and using its adenylate kinase activity to phosphorylate LDHA at Y10. Fibroblast growth factor receptor 1 (FGFR1) facilitates this process [107]. Multiple signaling pathways influence LDHA, modulating its activity and enhancing aggressive cancer trails. For example, FGFR1 phosphorylates LDHA at several tyrosine sites, differentially regulating LDHA and LDHB expression [109,110]. This regulation leads to increased LDHA stability through tyrosine phosphorylation and decreased LDHB expression through promoter methylation, driving a metabolic shift towards aerobic glycolysis. Receptor tyrosine kinases such as human epidermal growth factor receptor 2 (HER2) and Rous sarcoma oncogene (SRC) also activate LDHA via phosphorylation at tyrosine 10, resulting in higher migration, invasiveness, and metastatic potential in cancer cells [109,110].

Beyond direct phosphorylation by receptor tyrosine kinases, LDHA expression is also regulated by epigenetic mechanisms. For example, the histone demethylase jumonji domain-containing protein 2A (JMJD2A) promotes the Warburg effect in NPC by directly activating LDHA expression. JMJD2A binds to the LDHA promoter region, triggering the JMJD2A-LDHA signaling pathway and supporting NPC progression [50]. Additionally, LDHA plays a crucial role in promoting EMT and the development of cancer stem cell (CSC) properties, both essential for tumor growth and metastasis [64]. LDHA controls genes related to EMT, such as Snail, Slug, N-cadherin, E-cadherin, Vimentin, and Fibronectin, and also increases the expression of key stemness-related transcription factors like sex-determining region Y (SRY)-Box2 (SOX2), octamer-binding transcription factor 4 (Oct4), Nanog, and c-Myc [111]. This positive correlation between LDHA levels and CSC/EMT markers emphasizes its importance in driving tumor aggressiveness. Similarly, high levels of LDH-5 are associated with phosphorylated vascular endothelial growth factor receptor 2 (VEGFR2)/kinase insert domain receptor (KDR) expression, further linking LDHA activity with pro-angiogenic and metastatic processes [64].

Therefore, high levels of LDHA expression and phosphorylation positively influence cellular metabolism. EMT and CSC characteristics are essential drivers of tumor metastasis and progression. The intricate interaction among signaling pathways, epigenetic modifications, and LDHA activity emphasizes the complexity of cancer metabolism and identifies potential therapeutic targets to disrupt these processes.

### 3.2. Acetylation: Balance Between LDHA Activity and Degradation

Lysine acetylation also plays a crucial role in regulating LDHA activity. This process involves the recognition of acetylated lysine at position 5 (K5) in LDHA by the heat-shock cognate protein 70 (HSC70), which then transports the protein to lysosomes for degradation [112]. Therefore, K5 acetylation acts as a “tag” for LDHA breakdown, maintaining a balance between the enzyme’s activity and its level in the cell. In pancreatic cancer cells, a reduced lysine acetylation at K5 by deacetylase sirtuin 2 (SIRT2) results in increased LDHA enzymatic activity and decreased degradation [113]. Post-translational modifications, such as phosphorylation and acetylation, crucially regulate LDHA activity, enabling the cell to respond to changing conditions quickly. Phosphorylation, especially at Y10, boosts enzyme activity and promotes cancer progression. Conversely, K5 acetylation promotes LDHA degradation, helping to balance its activity and abundance in the cell. Understanding how post-translational modifications regulate LDHA provides new opportunities for developing therapies that target this enzyme and inhibit cancer cell growth.

## 4. LDHA Facilitates the Migration, Invasion, and Metastasis of Cancer Cells

### 4.1. LDHA in the Migration and Invasion of Cancer Cells

Many studies have highlighted the impact of LDHA on cancer cells’ ability to migrate and invade (Table 1) (Figure 1), making it a potential target for therapeutic intervention [114]. A survey of hepatoma cells showed that inhibiting LDHA nearly halved their motility [60]. This demonstrates a direct link between LDHA activity and the ability of cancer cells to migrate, which is essential for metastasis. Supporting this, research on CRC metastasis found a positive correlation between LDHA expression levels and the detection of distant metastases [115]. Further evidence comes from a study of hereditary leiomyomatosis (HL) and RCC, where cells with fumarase deficiency showed high invasiveness and lost this ability when LDHA was inhibited. This suggests that LDHA may be a key factor influencing cancer cell invasiveness in specific genetic contexts [116]. The primary way LDHA controls cell migration and invasion is through lactate production. Adding lactate from outside can enhance the random movement of tumor cells across different cancer cell lines [117,118]. The amount of lactic acid is closely linked to how often cancer spreads to distant sites [119,120,121]. Lactate influences metastasis through several key mechanisms, including activating matrix metalloproteinases (MMPs) and cathepsins due to the acidic environment it creates, increasing the regulation of VEGF, HIF1α, and transforming growth factor β2 (TGF-β2), and directly enhancing the cell’s ability to migrate [122,123,124,125]. Besides its effects via lactate, LDHA also controls the production of proteins involved in migration and invasion [2]. LDHA affects the behavior of the actin cytoskeleton, which is essential for cell movement. Research shows that LDHA interacts with cytoskeletal proteins, helping organize them and promoting the formation of lamellipodia and filopodia, known as structures crucial for movement [126,127,128]. LDHA also supplies energy for migrating cells because processes involving actin and other cytoskeletal components require ATP [129]. The activity of LDHA helps cancer cells stay energized for migration by maintaining the redox balance, converting pyruvate to lactate, and regenerating NAD^+^, which is needed for continued glycolysis [130]. Although less efficient than OXPHOS, active glycolysis allows cancer cells to produce ATP quickly, which is vital for migrating cells. Additionally, LDHA activates signaling pathways like phosphatidylinositol 3-Kinase (PI3K)/Akt and mitogen-activated protein kinases (MAPK) [24,131] and promotes the expression of genes encoding proteins such as N-cadherin, vimentin, and MMPs [60], all important for regulating cell migration. This regulation occurs through various pathways, including HIF1α, often activated in cancer cells. Under low oxygen conditions, HIF1α increases the expression of Snail, leading to higher levels of vimentin and N-cadherin and reduced levels of E-cadherin. These changes facilitate EMT and promote cell movement [132].

Studies have demonstrated that LDHA can regulate the expression of TGF-β2 in GBM. Specifically, an increase in LDHA levels results in higher TGF-β2 expression, leading to an increase in MMP-2, which then promotes extracellular matrix (ECM) degradation, aiding glioma cell migration. Conversely, reducing LDHA expression causes a decline in TGF-β2 protein levels and subsequently decreases cell migration [122]. This highlights the essential role of LDHA in controlling TGF-β2-driven glioma cell migration. Additionally, it has been shown that suppressing LDHA expression decreases the levels of focal adhesion kinase (FAK), MMP-2, and VEGF [122].

Thus, LDHA plays a multifaceted role in the migration and invasion of cancer cells (Figure 1), both through the production of lactate and the creation of a favorable microenvironment, and through the regulation of the expression of key metabolic genes.

### 4.2. LDHA and Cancer Cell Metastasis

Recent studies have highlighted the critical role of adhesion molecules such as intercellular adhesion molecule-1 (ICAM-1) and vascular cell adhesion molecule-1 (VCAM-1) in CTC biology. For example, ICAM-1 has been shown to promote CTC cluster formation and facilitate trans-endothelial migration, emphasizing its importance for metastatic spread [133,134]. Reviews on CTC mechanobiology further emphasize that adhesion molecules are crucial for CTC survival, interaction with blood components, and colonization of distant tissues [135]. Parallel evidence from tumor biology indicates that LDHA-derived lactate can regulate adhesion molecule expression: lactate signaling through GPR81 induces ICAM-1 in inflammatory settings, while HKla upregulates VCAM-1 transcription and fosters metastatic behavior [136,137]. Although direct experimental proof of LDHA-mediated regulation of ICAM-1 and VCAM-1 in CTCs is not yet available, the convergence of data linking LDHA activity, lactate signaling, and adhesion molecule expression supports the idea that metabolic reprogramming via LDHA may modulate adhesion pathways in CTCs to enhance their survival and metastatic potential. Testing this hypothesis in patient-derived CTCs could offer new insights into metabolic-adhesive interactions during metastasis.

LDHA also plays a vital role in maintaining redox homeostasis in CTCs, especially under the stress of detachment (anoikis). Detachment from ECM increases ROS, which can damage DNA, mitochondria, and activate intrinsic apoptotic pathways. By converting pyruvate to lactate and regenerating NAD^+^, LDHA supports antioxidant systems (e.g., glutathione, superoxide dismutase) and reduces ROS generation, helping CTCs survive in circulation [107,138,139,140]. Furthermore, LDHA activation is linked to PI3K/Akt signaling, which suppresses apoptosis and promotes proliferation under anoikis-like conditions. For example, in trophoblastic HTR-8/SVneo cells, knocking down LDHA decreases p-PI3K and p-Akt levels, reduces Cyclin D1, and increases apoptosis [24]. In other cancer cell lines, inhibiting LDHA enhances mitochondrial outer membrane permeabilization, increases VDAC activity, shifts the Bcl-2/Bax ratio toward apoptosis, triggers the release of cytochrome c, and activates caspases [141,142]. Collectively, these findings suggest that in CTCs, LDHA may support survival by reducing ROS and activating the PI3K/Akt and anti-apoptotic Bcl-2 family pathways. However, this hypothesis still requires direct testing in isolated CTCs from patients.

Thus, LDHA plays a vital role in maintaining the energy balance necessary for tumor spread, providing cancer cells with the energy required for migration, invasion, breaking down the ECM, and adapting to new microenvironments. Inhibiting LDHA activity is a promising therapeutic strategy to prevent cancer invasion and metastasis. Understanding LDHA’s role in supporting cancer metabolism opens new opportunities for developing more effective treatments.

## 5. Mechanisms of Action of Sodium Oxamate

### 5.1. Pharmacological and Molecular Aspects of LDHA Inhibition by Sodium Oxamate

The inhibitory mechanism of oxamate is based on its structural resemblance to pyruvate, as both molecules share a carboxylate group, while oxamate contains an amide substituent in place of the methyl group. This structural similarity enables oxamate to act as a competitive inhibitor by occupying the active site of LDHA [10]. Although glycolysis can proceed in normal aerobic cells with limited dependence on LDHA due to mitochondrial oxidative metabolism, aerobic glycolysis in cancer cells (the Warburg effect) is critically dependent on LDHA activity for continuous NAD^+^ regeneration and sustained glycolytic flux [11,12]. Under hypoxic conditions, LDHA also plays an essential role in maintaining glycolysis in normal cells by facilitating adaptation to reduced oxygen availability [13]. In malignant cells, however, LDHA is frequently overexpressed and constitutively active, supporting high glycolytic rates irrespective of oxygen levels [14]. This persistent activation enables cancer cells to maintain elevated glycolytic flux, which can exceed that of normal cells by an order of magnitude, thereby promoting rapid ATP generation and biosynthetic precursor production required for tumor growth [15]. Accordingly, LDHA is widely recognized as a central regulator of glycolytic metabolism and a key determinant of metabolic reprogramming in cancer.

Crystallographic data show that oxamate binds to the active site of LDHA, forming a non-condensed complex with the enzyme, which prevents the normal conversion of pyruvate to lactate [143]. As a result, lactate synthesis in cells decreases, and the NAD^+^ regeneration process is disrupted, limiting further glycolysis and ATP production [144]. An additional mechanism by which oxamate and other LDHA inhibitors may suppress tumor growth is by blocking lactate reutilization. In oxygenated tumor regions, lactate can be converted back to pyruvate via LDH-A/B and funneled into OXPHOS. Inhibiting this pathway deprives cancer cells of an important oxidative fuel, limiting metabolic flexibility and growth [78].

In an experiment with HeLa mitochondria, oxamate reduced lactate production, indicating a decrease in the reaction converting pyruvate to lactate [145]. In cellular models of human gastric cancer cell line SGC7901 [17], NPC [28,29], invasive pituitary adenomas, medulloblastomas, GBMs [87], pituitary adenoma [146] and renal proximal tubular cells (NRK-52E) [144], oxamate promoted pyruvate accumulation and decreased NAD^+^ and ATP levels, which triggered compensation through OXPHOS. Activation of mitochondrial respiration results in ROS buildup, leading to cell death via apoptosis or autophagy. T-cell acute lymphoblastic leukemia (T-ALL) is a rare and highly aggressive form of ALL. Treatment of the major T-ALL cell lines, Jurkat and DU528, with the LDH inhibitor oxamate resulted in a pronounced reduction in cell proliferation and induction of apoptosis. Oxamate also caused cell-cycle arrest at the G0/G1 phase and significantly increased intracellular ROS levels. Pharmacological LDHA inhibition led to a substantial decrease in both mRNA and protein expression of c-Myc, as well as reduced phosphorylation of AKT and GSK-3β within the PI3K signaling pathway. Consistently, genetic silencing of LDHA in a T-ALL transgenic zebrafish model delayed disease progression and downregulated c-Myc expression at both the transcript and protein levels [44]. Collectively, these findings demonstrate that oxamate inhibits LDHA through competitive active-site binding, suppressing glycolysis, disrupting NAD^+^ regeneration, and forcing a metabolic shift toward OXPHOS and ROS accumulation. This metabolic stress reduces proliferation, induces apoptosis or autophagy, and downregulates oncogenic pathways such as c-Myc and PI3K/Akt, highlighting LDHA inhibition as a promising anticancer strategy across diverse tumor models.

### 5.2. Impact on Signaling Pathways and Cellular Functions

In addition to directly inhibiting LDHA activity, oxamate also impacts signaling pathways that regulate cell viability. The mammalian target of rapamycin (mTOR) is a protein kinase that regulates key cellular processes such as metabolism, catabolism, immune function, autophagy, cell survival, proliferation, and migration, thereby ensuring cellular homeostasis [147,148]. Besides suppressing glycolysis, oxamate can inhibit the Akt-mechanistic target of the mTOR pathway, which reduces cell growth and promotes autophagy. In the NSCLC cell line A549, it has been demonstrated that oxamate reduces levels of p-Akt and p-mTOR, triggering protective autophagy, and blocking this process with 3-methyladenine switches cells to apoptosis [20]. This directly confirms the influence of oxamate on the Akt-mTOR signaling pathway. Oxamate decreases standard glycolysis, leading to lower ATP and NAD^+^ levels, with subsequent metabolic compensation via OXPHOS, an increase in oxygen consumption rate (OCR), especially when combined with phenformin. When combined with rapamycin, oxamate results in a greater suppression of glycolysis and growth in CRC cells, demonstrating a synergistic effect that involves inhibiting LDHA and mTOR activity [35]. In medulloblastoma cells, oxamate inhibits glycolysis and activates OXPHOS, leading to reduced proliferation due to cell cycle arrest in the G2/M phase [43]. In gastric cancer (SGC7901, BGC823) and HCC (Huh7, PLC), inhibiting glycolysis with oxamate increases E-cadherin expression and decreases vimentin, Snail, and fibronectin levels, which are indicators of EMT inhibition [17,149]. In gastric cancer (SGC7901) cells, oxamate suppresses phosphorylation of Akt and mTOR, activating autophagy. Inhibiting autophagy with chloroquine or Beclin1 small interfering ribonucleic acid (siRNA) enhances apoptosis. This clearly demonstrates that oxamate influences key signaling pathways, not just glycolysis.

Simultaneously, a change in the cell’s energy profile after LDHA blockade can lead to the reprogramming of metabolic processes, which contributes to the emergence of resistant subpopulations that utilize alternative pathways. While oxamate effectively inhibits glycolysis and can suppress tumor growth in many cell types, some cells develop resistance mechanisms, enabling them to survive and proliferate even when LDH is blocked [150]. This resistance often involves metabolic reprogramming, such as shifting to glutamine metabolism [151] or utilizing other LDH isoforms [152], highlighting the complexity of targeting cancer metabolism (described in detail in Section 7.3 Relationship with metabolic plasticity).

## 6. The Influence of Oxamate on Migration, Invasion, and Metastasis

**Figure 2:**
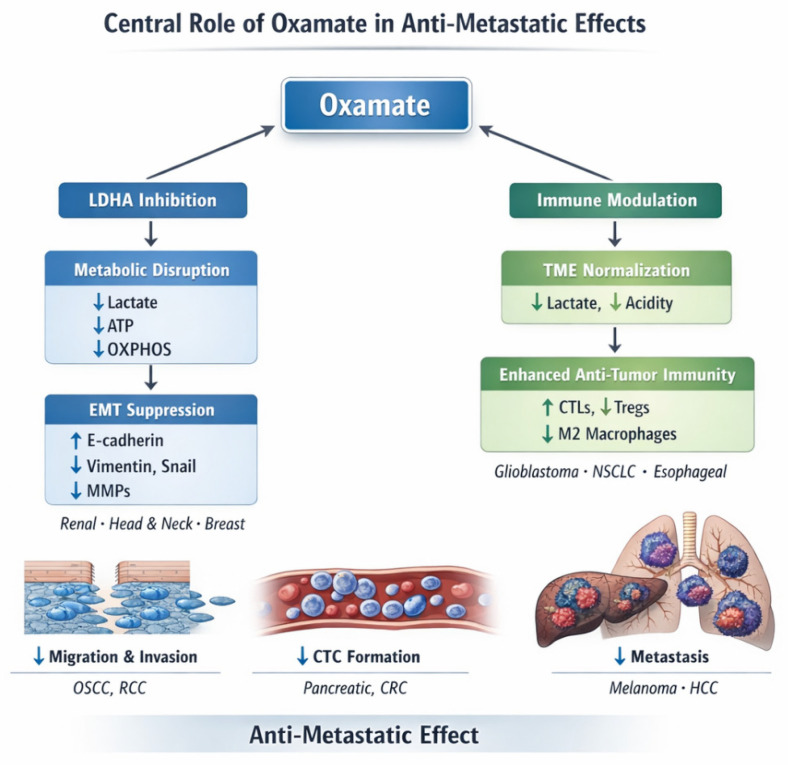
Oxamate inhibits LDHA to drive metabolic reprogramming and suppress metastatic phenotypes in cancer. Schematic illustration of the mechanistic cascade of oxamate action: as a competitive inhibitor of LDHA, oxamate reduces LDHA enzymatic activity, thereby decreasing glycolytic flux and lactate production. This inhibition induces downstream metabolic perturbations, including pyruvate accumulation, depletion of NAD^+^ and ATP, and compensatory upregulation of mitochondrial OXPHOS. The shift toward OXPHOS elevates mitochondrial ROS levels, which triggers cancer cell apoptosis or autophagy. Across multiple preclinical cancer models, oxamate treatment diminishes cancer cell proliferation, migration, and invasive capacity, attenuates EMT features, and enhances sensitivity to anti-cancer therapies, highlighting the potential of oxamate as a metabolic adjuvant to improve anti-metastatic and therapeutic outcomes.

One of the most notable effects of oxamate is a reduction in the ability of cells to migrate and invade. Studies in OSCC found that inhibiting LDHA with oxamate decreases cell migration. This is linked to lower lactate production, which affects the acidity of the microenvironment and cell motility [30]. Oxamate reduced the migration and invasion of the 786-O cell line, derived from a primary clear cell renal cell carcinoma (ccRCC), in transwell and scratch assays. This was associated with reduced LDHA activity, glucose metabolism, and lactate production in culture [36]. Additionally, the oxamate-mediated downregulation of LDHA impairs tumor necrosis factor alpha (TNF-α)-dependent esophageal cancer cell migration and reduces TNF-α-induced MMP9 expression [18]. Pretreatment of Lewis lung carcinoma (LLC) cells with oxamate under adherent conditions reduced the viability of these cells when they were transferred to anchorage-independent growth conditions, which serve as a model for cancer cells that circulate freely in the blood [26]. Thus, oxamate directly inhibits the motility and invasion of various cancer cells, like NSCL [27], NPC [28,29], oral squamous cell carcinoma (OSCC) [30], esophageal [18], gastric [31], pancreatic [32], HCC [33,34], CRC [35], RCC [36], MIBC [37], melanoma [38], cervical [39], ovarian [40], breast [41], prostate cancer [42], medulloblastoma [43], GBM [19], and T-ALL [44], by lowering lactate production, environmental acidity, the expression of EMT markers, and MMP enzymes [4,27,46] (Table 2 and Figure 2). It also decreases the activity of signaling pathways, such as PI3K/Akt, that are involved in migration and angiogenesis.

## 7. Metabolic Plasticity and Connection to EMT

### 7.1. Oxamate-Mediated Control of EMT in Metastasis

EMT is a process where cells lose their epithelial traits, like cell adhesion, and gain mesenchymal features, enabling them to migrate and invade nearby tissues. EMT activity is closely linked to the expression of proteins such as E-cadherin, N-cadherin, vimentin, cytoskeletal remodeling, and the activation of TGFβ, Wnt, and Notch signaling pathways. This enhances the cells’ ability to infiltrate tissues and metastasize [153,154,155,156,157]. Studies have shown that EMT promotes the formation of CTCs and their capacity to enter the bloodstream, which is vital for the hematogenous and lymphatic spread of tumors [158]. Research into the molecular mechanisms of EMT indicates that the loss of E-cadherin and the activation of Snail/ZEB/Twist factors contribute to changes in cell adhesion and polarity, including pseudopodia formation and directed migration [159]. The link between EMT and metastasis is well established. In head and neck squamous cell carcinoma (HNSCC), low E-cadherin or high vimentin levels are associated with a higher risk of metastasis [160]. Recent findings suggest that EMT triggers the formation of mesenchymal stem cells that can migrate, cause metastases, and exhibit resistance to therapy [161,162]. Another study demonstrated that EMT initiates the loss of cell contacts, reprograms the cytoskeleton, and activates MMP expression, all promoting invasion and metastatic foci formation [163]. In summary, EMT activation involves the loss of E-cadherin, increased expression of vimentin and N-cadherin, and heightened MMP activity, leading to enhanced cell mobility, invasion, CTC formation, and colonization of new tissues.

Studies highlight the role of vimentin as a key marker and driver of EMT. Its expression signals the activation of migration-related genes, ECM breakdown, angiogenesis, and therapy resistance [164]. Vimentin is not just a marker of EMT but also actively participates in migration by interacting with PI3K/Akt pathways and affecting the cytoskeleton and β-catenin signaling [165]. Using oxamate decreases vimentin expression by inhibiting LDHA. A study on the 786-O cell line (ccRCC) found that oxamate reduces LDHA activity, lactate production, and glucose consumption [36]. Oxamate decreased the expression of EMT markers (Figure 2) such as Snail, vimentin, MMP2, and MMP9 in the cell ccRCC [36]. This also reverses EMT, as shown by increased E-cadherin, decreased vimentin, and the transcription factor Snail, which leads to reduced migration and invasion. Similar results were observed in OSSC cells. In HSC3 and SCC15 cultures, LDHA inhibition with oxamate lowered glycolytic activity and cell growth, and suppressed migration, invasion, and tumor nodes [30]. By inhibiting LDHA, oxamate causes metabolic stress, decreases lactate, NAD^+^, and ATP, and boosts OXPHOS and ROS production. This enhances effects on Akt-mTOR signaling and reduces EMT markers, including vimentin [166]. Oxamate promotes the reduction in vimentin levels by altering energy metabolism and suppressing key signaling pathways regulated through metabolic changes, especially via the Akt-mTOR pathway.

The link between lactate and histone modifications that induce EMT is emphasized. A mouse study examined the role of lactate and HKla in activating EMT in diabetic nephropathy (DN) [167]. It was observed that during chronic hyperglycemia, elevated lactate levels trigger HKla. This results in a decrease in the expression of epithelial markers like nephrin and zonula occludens-1, while increasing the expression of mesenchymal markers such as smooth muscle actin alpha and collagen IV, thereby contributing to the pathological development of DN. Glycolysis inhibitors (oxamate, DCA) block this process, lowering HKla and EMT markers both in vitro and in vivo [167].

Consequently, oxamate exerts a complex inhibitory effect on EMT (Figure 2) by suppressing key metabolic and epigenetic drivers through (i) inhibiting LDHA and reducing lactate levels, (ii) promoting a shift from mesenchymal to epithelial cell states, (iii) decreasing cell migration and invasion, and reducing metastasis and tumor growth in animal models, and (iv) regulating epigenetics by neutralizing lactate’s effects on histone modification. Together, these findings demonstrate the potent anti-metastatic effect of oxamate by modulating EMT, metabolism, and epigenetic mechanisms.

### 7.2. Systemic and Immunomodulatory Effects of Oxamate

Lactate plays a key role in immunosuppression within the TME. Its excessive production by tumor cells results from enhanced glycolysis (Warburg effect), leading to local lactate buildup and acidification of the surrounding environment. This causes various immunosuppressive effects, as confirmed by numerous experimental and clinical studies and well-described in recent reviews [168,169,170,171,172,173]. Beyond its well-characterized metabolic effects, oxamate elicits immunomodulatory activity within the TME, where it reverses lactate-driven immunosuppression, modulates myeloid cell polarization, and enhances anti-tumor T-cell function—processes that collectively contribute to its anti-metastatic potential [6,16]. Elevated lactate levels in the TME have been shown to suppress CTL activity and promote regulatory T cell (Treg) function, thereby facilitating immune evasion [17]. By inhibiting LDHA and reducing lactate production, oxamate alleviates these immunosuppressive effects and restores anti-tumor immune responses. Notably, the immunomodulatory effects of oxamate appear to be largely secondary to its inhibition of tumor-derived lactate production. Lactate has been demonstrated to regulate immune cell function through metabolic and epigenetic mechanisms, including HKla and modulation of macrophage polarization [5]. However, the extent to which oxamate exerts direct, lactate-independent immunoregulatory effects remains insufficiently characterized, and current evidence predominantly supports an indirect mechanism mediated through metabolic reprogramming of the TME.

In GBM, high lactate levels are linked to blocked infiltration of CTLs and immune suppression [174]. Oxamate restores the functionality of CTLs within the tumor. In GBM, oxamate decreases lactate concentration in the tumor environment, reduces HKla (H3K18la) on promoters of crucial immunoregulatory genes like CD39, CD73, and CCR8, and lowers their expression on intra-tumoral Treg cells, thereby decreasing their accumulation and activity [174]. Weakened immunosuppression enhances the protective function of chimeric antigen receptor-T (CAR-T) cells, which increases the production of interferon gamma (IFNγ), perforin, and granzyme B, thereby improving the immune response against the tumor [175]. Studies using the humanized mouse model of NSCLC also show that oxamate reduces lactate production and TME acidity. This promotes increased infiltration of activated CTLs and boosts the effectiveness of pembrolizumab in blocking the programmed cell death protein-1 (PD1) receptor on T-cell surfaces, enabling the immune system to attack cancer cells more effectively [27]. Similar synergies between PD1 inhibition and immunomodulatory agents have been observed with nanoparticle (NP) tumor vaccines, which enhance T-cell activation and clear CTCs to further suppress metastasis [176]. In the TE1 esophageal cancer model, lactate released by tumors stimulates macrophage polarization into an immunosuppressive M2 phenotype, increasing arginase-1 (Arg-1) activity and mannose receptor C-type 1 expression through the Akt/extracellular signal-regulated kinase (ERK) pathway. Oxamate inhibits these effects by reducing lactate levels, Akt/ERK activation, and M2 polarization both in vitro and in vivo [177]. Similarly, in a gastric cancer model, oxamate contributes to inhibiting LDHA, decreasing lactate production, and preventing M2 macrophage polarization via the MCT-HIF1α pathway [178]. In the LLC model, a dose-dependent effect on peritoneal macrophages was observed. At low doses of oxamate, M2 macrophage markers were activated, Arg-1 activity increased, and NO synthase activity decreased. Conversely, high doses of oxamate caused macrophages to adopt an M1 polarization, increasing NO synthase activity and decreasing Arg-1 activity [179]. This suggests potential context-dependent immunomodulatory effects of oxamate on macrophages.

Thus, oxamate in the TME decreases lactate production and normalizes pH, epigenetically suppresses key immunoregulatory genes, reduces immune suppression (Treg, M2), and enhances the activity of tumor-specific immunity (CAR-T cells, CTLs). However, a double response by macrophages is possible, which is dose-dependent polarization toward M1 or M2. A comprehensive understanding of these dynamics is essential for optimizing therapeutic strategies. Overall, the effects of oxamate demonstrate how metabolic changes can directly enhance the immune response. Such a shift in the immune environment reduces tumor cell proliferation and diminishes their ability to migrate and invade, as immune normalization can limit local invasion and metastasis formation.

### 7.3. Relationship with Metabolic Plasticity

Tumor cells develop resistance to LDHA inhibitors via key adaptive mechanisms, including compensatory upregulation of LDHB to sustain lactate metabolism and a metabolic shift toward a more oxidative phenotype. This latter adaptation may reduce proliferative rates due to diminished glycolysis-derived anabolic intermediates but fails to eliminate tumor cells, representing a critical therapeutic challenge [141]. Metabolic plasticity is a key feature of tumor cells, enabling them to adapt to changing microenvironmental conditions such as oxygen deprivation, nutrient deficiency, or therapeutic stress. When one pathway is blocked, cells can activate alternative energy routes to sustain survival and growth. Several studies have demonstrated that blocking or knocking down LDHA alone often prompts metabolic compensation through other LDH isozymes, especially LDHB. This compensation allows cancer cells to sustain lactate production, glycolytic flux, or at least survive by shifting toward oxidative phosphorylation. For example, a double knockout of LDHA and LDHB is necessary to eliminate the Warburg effect and force cells in colon and melanoma lines to rely on OXPHOS as the main ATP source, whereas knocking out LDHA alone still leaves residual LDH activity likely mediated by LDHB [152]. Studies in GBM have shown that individual CRISPR-mediated knockout of either LDHA or LDHB has only a limited effect, whereas double LDHA/LDHB knockout induces a profound metabolic reprogramming characterized by enhanced OXPHOS and reduced tumor growth [180]. Neuroblastoma research indicates that high LDHA expression is associated with poorer prognosis; depletion of LDHA reduces neuroblastoma growth in vitro and in vivo, yet LDHB remains expressed in the same cells, which helps explain the residual LDH activity and why the impact on aerobic glycolysis does not always directly correlate with the anticancer effect [9,181]. Similarly, in medulloblastoma cells, LDHA silencing was less effective than broad LDH inhibition (e.g., with oxamate), indicating that LDHB and other LDH family members compensate when LDHA is absent. In neuroblastoma, depleting LDHA reduces tumor growth, but LDHB is still expressed in the same cells and contributes to remaining LDH activity, highlighting redundancy among LDH isozymes [43]. A study in LLC cells tested the effects of oxamate under glutamine-deficient conditions. Glutamine deficiency increased the cytotoxic effects of oxamate on LLC cells. The study found that some cell subpopulations were resistant to oxamate, but many became sensitive under glutamine-deficient conditions. These surviving cells apparently switched to using glutamine as an alternative energy source through activation of glutaminolysis [182]. Lastly, recent studies (e.g., in cervical or pancreatic cancer) suggest that LDHA inhibition can also activate mitochondrial pathways and AMPK/mTOR-S6K signaling, enabling cells to rewire their metabolism and resist LDHA-targeted therapy [183].

Taken together, these findings emphasize that tumor cell survival depends on alternative energy pathways. Therefore, an effective treatment approach should target multiple metabolic pathways at once, including glycolysis, glutaminolysis, and mitochondrial function, to prevent compensatory mechanisms and ensure sustained tumor suppression.

## 8. Clinical Perspectives and Synergistic Approaches in Therapy

### 8.1. Current Clinical Trial Data

Although most studies on oxamate have been conducted in animal models or in vitro, the data obtained demonstrate significant potential for using this inhibitor in clinical practice. One promising area is the combined use of oxamate with drugs targeting both mitochondrial and cytoplasmic components. A study examining the combined use of phenformin and oxamate revealed that phenformin, an inhibitor of mitochondrial complex I, together with oxamate, enhances the effects of reducing cell viability, decreasing lactate production, and maintaining ROS levels, leading to a significant increase in tumor cell apoptosis [148]. In preclinical studies involving an NSCLC model in humanized mice, oxamate slowed tumor growth, increased infiltration of activated CTLs, and, when combined with pembrolizumab, produced a much stronger effect [27]. A study in a GBM model showed that oxamate enhances the efficacy of CAR-T therapy, reduces the expression of immunoregulatory molecules (CD39, CD73, CCR8) in Treg cells, and boosts the activity of CD8^+^ tumor-infiltrating lymphocytes (TILs) and CAR-T cells, thereby reducing tumor growth and increasing animal survival [175]. Oxamate also increases the sensitivity of GBM and medulloblastoma to temozolomide and radiotherapy by triggering ROS-mediated mitochondrial apoptosis, which prevents tumor growth [184].

The data obtained confirm the potential of oxamate in treating aggressive central nervous system tumors. In a triple combination model (doxorubicin + metformin + oxamate), the glycolysis and TCA cycle inhibition blocked mTOR activity, reduced HIF1α, and eliminated tumor nodes in mice with colorectal tumors. Additionally, the triple combination promotes the activation of apoptosis, autophagy, and complete healing in vivo without significant toxicity [185]. Analogs of LDHA inhibitors CHK336 (Novartis/Chinook) have completed Phase I clinical trials (clinical trial number NCT05367661), confirming acceptable tolerability and safety in patients with solid tumors. Dose-dependent activity was observed, including partial responses in some patients. The maximum tolerated dose was established, paving the way for further Phase II studies [186,187]. These data indicate the potential efficacy of LDH inhibitors in oncology, particularly in tumors with high glycolytic activity.

### 8.2. Problems and Prospects for Implementation

Oxamate is an effective LDHA inhibitor, but its high polarity and low permeability through cell membranes remain significant obstacles to clinical use. These issues are mainly related to its chemical and pharmacokinetic properties. The polarity of oxamate results from the presence of carboxyl and amide groups [188]. These groups ionize at physiological pH, which reduces their ability to passively diffuse through lipid cell membranes [189]. As a result, low cellular permeability limits its effectiveness in systemic therapy. Due to its hydrophilicity, oxamate can be rapidly excreted from the body through the kidneys [190], leading to a short plasma half-life and the need for frequent or higher doses to achieve a therapeutic effect. In the body, oxamate undergoes rapid metabolism, although the specific metabolic pathway in humans is not fully understood. This may also reduce its effectiveness when administered systemically. Unlike some pyruvate or lactate analogs [191], oxamate is not taken up by specialized membrane transporters. Oxamate is routinely used at 10–200 mM in vitro and in vivo across dozens of published studies (Table 2), with 20–60 mM representing the standard working range for migration/invasion assays, 10–20 mM eliciting mild to moderate LDHA inhibition, 50–100 mM driving strong inhibition [146], and 100–200 mM achieving maximal effects [32] (particularly in in vivo models, where lower concentrations are functionally ineffective). This high concentration requirement is a well-documented challenge directly attributable to three key factors: oxamate’s high polarity and poor cellular membrane permeability, the need for excess inhibitor to compete with intracellular pyruvate for the LDHA active site, and tumor cell metabolic plasticity, wherein cells compensate for LDHA inhibition via upregulation of oxidative phosphorylation and glutaminolysis.

In recent years, the rapid development of nanomedicine has provided a transformative solution to overcome the clinical barriers of oxamate and other poorly permeable therapeutic agents. Contemporary studies have demonstrated that NPs are successfully used for both molecular imaging [192,193,194], and targeted drug delivery [195,196], showing high efficacy in preclinical and clinical models of tumor growth. NPs, typically ranging in size from 10 to 200 nm, possess unique physicochemical properties that make them ideal drug carriers: their small size allows for passive targeting to tumor tissues via the enhanced permeability and retention effect [197,198], while their surface can be easily functionalized with ligands [199,200,201], antibodies [176,202], or peptides [203,204] to achieve active targeting of [205]. Moreover, NPs can encapsulate hydrophilic drugs such as oxamate within their core, shielding their polar groups and facilitating transmembrane transport through endocytosis, thereby significantly improving intracellular drug delivery efficiency. The anti-GD2 immunoliposome system, specific for neuroblastoma and loaded with oxamate, has been successfully used, providing targeted delivery and reducing effects on healthy tissues, thereby increasing oxamate concentration in tumors [202]. A prodrug based on SN22-tocopheryl oxamate, encapsulated in polylactic polyethylene glycol liposomes, has also been developed, which in mice with chemoresistant neuroblastomas caused rapid tumor regression and prolonged survival [206]. Successful incorporation of oxamate with cyclophosphamide into niosomes was demonstrated (~88 nm in size) [196]. Charged niosomes provided stable encapsulation. In MDAMB231 (triple-negative breast cancer), they increased apoptosis by elevating Bax and caspase-3 expression and reducing cell viability. The effectiveness of niosomes significantly exceeded that of free oxamate [196]. Recent studies have developed oxamate NPs using the thin-layer hydration method to improve delivery to tumor cells. The NPs were effectively taken up by tumor cells via endocytosis. Once inside, they release oxamate ions (NH_2_COCOO^−^) and sodium ions (Na^+^) [195]. This release also causes osmotic and oxidative stress, which can promote the development of pyroptosis. The use of these NPs reduced the proliferation, migration, and invasion of tumor cells, and also exerted an immunomodulatory effect in the TME. Thus, nano-carriers with oxamate demonstrate substantially better anti-tumor effects in vitro and in vivo compared to the free drug.

The use of NPs opens new opportunities to enhance the bioavailability and therapeutic efficacy of tumor cell energy metabolism blockers, particularly glycolysis inhibitors such as oxamate. Encapsulation or conjugation of metabolic inhibitors with NPs improves their pharmacokinetic properties, reduces systemic toxicity, and ensures selective accumulation within tumor tissue and metastatic lesions [207,208]. In addition, functionalized NPs can combine therapeutic and diagnostic properties (the theranostic approach), enabling simultaneous treatment and real-time monitoring of drug distribution. Incorporating fluorescent, magnetic, or radioactive labels into NPs or using quantum dots allows tracking of their localization in primary tumors and metastases [209], as well as assessment of intratumoral heterogeneity in drug accumulation [92]. Particularly promising is the application of NPs for visualization and analysis of their distribution at the single-cell level, including circulating and de-adherent tumor cells [176,210].

Such an approach provides opportunities not only to control the delivery of energy metabolism inhibitors but also to perform functional evaluation of the metabolic state of tumor cells, including glycolytic activity, oxidative phosphorylation, and associated signaling pathways. In the context of metastasis, this is especially important, as it enables investigation of metabolic plasticity, resistance to anoikis, and cellular adaptation to stressful microenvironmental conditions. Thus, integration of NPs into strategies for delivering metabolic inhibitors, including oxamate, holds significant potential for enhancing antitumor therapy efficacy and for deepening our understanding of the metabolic mechanisms underlying tumor progression and metastasis.

## 9. Conclusions

Preclinical evidence confirms that oxamate-mediated LDHA inhibition exerts context-specific, multi-dimensional effects on tumor progression. By reducing tumor-derived lactate, oxamate rewires cancer cell metabolism, normalizes the acidic TME, and suppresses EMT, thereby attenuating cancer cell migration, invasion, and metastatic spread. Crucially, oxamate also exhibits distinct immunomodulatory activity by reversing lactate-driven immunosuppression and reshaping myeloid cell polarization within the TME. This double metabolic–immune mechanism distinguishes it from single-action agents and supports its potential as a combinatorial partner to enhance the efficacy of chemotherapy and immunotherapy. Key translational challenges remain, including oxamate’s high polarity and tumor cells’ adaptive metabolic plasticity, which necessitate innovative delivery strategies and rational combination regimens to circumvent resistance. Collectively, oxamate represents a promising LDHA-targeted therapeutic prototype with the capacity to co-target metabolism, EMT, and immune evasion, offering a viable strategy to address the unmet clinical need in treating metastatic malignancies.

## Figures and Tables

**Table 1:** LDHA and metastasis: Biological effect and cancer types.

Cancer Types	Target/Mechanism	Effect of LDHA	Reference(s)
Non-small-cell lung cancer (NSCLC)	LDHA overexpression reduced CD8^+^ T cell infiltration	Promote the therapy resistance	[51]
Papillary thyroid carcinoma (PTC)	LDHA increases the gene H3K27 acetylation	Induces EMT, regulates autophagy, and promotes metastasis	[52]
Nasopharyngeal carcinoma (NPC)	LDHA-dependent glycolytic shift	Promotes progression by the TAK1/NF-κB Axis, production of lactate, growth, and invasion	[53,54]
Esophageal cancer	LDHA in TNF-α-induced cell migration by the ERK1/2 signaling pathway	LDHA inhibition suppresses TNF-α-driven migration	[18]
Gastric cancer	FOXM1 promotes LDHA expression	Induces protective autophagy, promotes the Akt-mTOR signaling pathway	[31,55]
Pancreatic adenocarcinoma	LDHA-activated aerobic glycolysis	Induced resistance to chemotherapy, metastasis, and transcription through the Wnt/β-catenin signaling pathway.	[56,57,58,59]
Hepatocellular carcinoma (HCC)	LDHA increases lactate secretion and anaerobic metabolism	Promote tumorigenesis and metastasis in vivo	[33,60]
Colorectal cancer (CRC)	REG1α via β-catenin/MYC activated LDHA	Drives metabolic reprogramming and metastasis	[61]
Renal cell carcinoma (RCC)	LDHA promotes EMT	Enhances migration, invasion; LDHA inhibition reduces metastasis	[36]
Muscle invasive bladder cancer (MIBC)	LDHA-induced anaerobic metabolism, LDHA targeting through miR-200c	Increases stem cell markers and poor outcome. Correlates with proliferation, invasion, and metastasis	[62,63]
Endometrial cancer	LDHA-dependent glycolytic shift	activated VEGF/VEGFR2 pathway	[64]
Melanoma	LDHA-dependent lactate accumulation and immune suppression	Suppresses T/NK cell activity; linked to metastasis and poor prognosis	[65]
Cervical cancer	Nuclear LDHA, α-HB/ROS interaction	Nuclear LDHA promotes cancer progression by producing the antioxidant α-hydroxybutyrate (α-HB) from elevated ROS	[8]
Ovarian cancer	LDHA downregulated by knockdown special AT-rich-binding protein 1 (SATB1)	Reprogramming energy metabolism increases proliferation and metastasis	[66]
Breast cancer	LDHA increases lactate secretion	Lactate promotes evasion of hypoxia-induced cell cycle arrest	[67]
Prostate cancer	KAT2A succinylates LDHA	Increases migration and invasion	[68]
Glioblastoma (GBM)	Inhibition of LDHA	Enhanced radiation sensitivity and sensitized cells to Temozolomide	[69]
Mechanistic study	LDHA inhibition (oxamate/siRNA) → ↓ lactate, ↑ pyruvate, shift to OXPHOS → ↑ ROS → apoptosis	Foundational evidence linking LDHA, metabolism, and tumor maintenance	[70]
Pan-cancer analysis	LDHA overexpression across cancers	Associated with aggressiveness, immune evasion, and poor prognosis	[45]

**Table 2:** Oxamate as an inhibitor of LDHA: Cancer types, mechanisms, and observed effects.

Cancer Types	LDHA-Related Target/Mechanism	Observations of the Effect of Oxamate (LDHA Inhibition)	Key Reference(s)
Non-small cell lung cancer (NSCLC)	LDHA supports glycolysis, survival under hypoxia	Oxamate suppresses proliferation, reduces ATP and NAD(P)H balance, causes ROS burst, G2/M arrest, and apoptosis in sensitive NSCLC lines. Enhances the Efficacy of Anti-PD-1 Treatment	[27]
Nasopharyngeal carcinoma (NPC)	LDHA-driven glycolysis	Oxamate suppresses NPC cell proliferation, Induces Apoptosis and G2/M Arrest increases radiosensitivity	[28,29]
Head & neck cancers (including OSCC)	LDHA drives EMT, migration, and invasion	Oxamate suppresses proliferation, migration, and motility in OSCC/head-neck models; it can sensitize to therapy	[30]
Esophageal cancer	LDHA contributes to TNF-α-induced migration	Oxamate inhibits MMP9 expression by affecting TNFα-induced ERK1/2 and thus suppresses tumor cell proliferation, migration, and invasion both in vitro and in vivo	[18]
Gastric cancer (SGC-7901, AGS)	LDHA increased glycolytic metabolism	Oxamate treatment → decreased lactate, increased pyruvate, decreased NAD^+^/ATP, induction of autophagy/apoptosis, reduced proliferation/migration	[31]
Pancreatic Cancer	Elevated expression of LDHA	Inhibition of LDHA by oxamate disrupts metabolic flux by reducing glucose uptake and altering levels of intermediate metabolites, thereby suppressing proliferation	[32]
Hepatocellular carcinoma (HCC)	LDHA increases lactate secretion and anaerobic metabolism	Promote tumorigenesis and metastasis in vivo	[33]
	LDHA activated lactate production, the YAP pathway, enhancing proliferation, migration, and invasion	Oxamate/LDHA suppression lowers lactate, reduces migration/invasion, and metastatic markers in HCC models	[34]
Colorectal cancer (CRC)	LDHA supports glycolytic flux and survival	Oxamate inhibition of LDH sensitizes colon cancer cells to rapamycin	[35]
Renal cell carcinoma (RCC)	LDHA promotes EMT and migration	Oxamate or LDHA knockdown blunts EMT markers (N-cadherin, vimentin), reduces motility	[36]
Bladder cancer (MIBC)	LDHA activated EMT and glycolytic support of invasion	Oxamate reduces invasion and glycolysis markers in bladder cancer models	[37]
Melanoma	LDHA enhances lactate accumulation and immunosuppression	Oxamate reduces lactate production and can restore antitumor immune function in preclinical models (improves T/NK cell activity)	[38]
Cervical cancer (HeLa and SiHa cells)	LDHA participated in cervical cancer pathogenesis	Oxamate reduces LDHA activity, blocks the cell cycle in the G2/M phase, decreases proliferation, and can trigger mitochondrial apoptosis in cervical cancer models	[39]
Ovarian cancer	LDHA enhances glycolysis, PARP activity	Oxamate synergized with PARP inhibitors, enhanced DNA damage, and suppressed tumor growth in vitro and in vivo	[40]
Breast cancer	LDHA increased glycolysis, alters the levels of E-cadherin, vimentin, p38 MAPK, ERK1/2, and AKT	Oxamate (or LDHA inhibition) reduces lactate, lowers proliferation, and invasiveness	[41]
Prostate cancer	LDHA activity supports proliferation, migration, invasion, and metastasis	Oxamate or LDHA targeting reduces glycolysis, decreases invasive behavior in LDH-dependent prostate models (mechanistic studies show metabolic vulnerability)	[42]
Medulloblastoma (MB)	High LDHA-activated glycolysis	Oxamate attenuates glycolysis, proliferation, and motility, and upregulates OXPHOS in MB cell lines	[43]
Glioblastoma (GBM)	LDHA supports the glycolytic phenotype and radio-resistance	Oxamate decreases EGFR expression, reduces cell stemness, modulates EMT markers, induces apoptosis, enhances radiosensitivity, and increases cell death in some GBM models	[19]
T-cell acute lymphoblastic leukemia (T-ALL)	LDHA increased disease progression and up-regulated c-Myc protein expression	Oxamate inhibited proliferation, induced arrested cells in the G0/G1 phase, stimulated ROS production, and apoptosis	[44]
General/Pan-cancer analysis	LDHA high expression correlates with poor prognosis, immune evasion and therapy resistance	Oxamate shows broad anti-glycolytic effects across many tumor types; pan-cancer analyses nominate LDHA as a biomarker/vulnerability, combination approaches (oxamate + biguanides/IO) are promising preclinically	[45]
Mechanistic summary	LDHA plays a key role in cancer cell metabolism to ensure rapid growth, survival and immune suppression	On-target inhibition of LDHA, including with oxamate, actually disrupts cancer cell metabolism (reduced glycolysis, lactate, ATP) and leads to a reduction in tumor growth in vivo, making LDHA and oxamate promising targets/agents in oncology	[4,46]

## Data Availability

No new data were created or analyzed in this study. Data sharing is not applicable to this article.
